# Associations between computed tomography markers of cerebral small vessel disease and hemorrhagic transformation after intravenous thrombolysis in acute ischemic stroke patients

**DOI:** 10.3389/fneur.2023.1144564

**Published:** 2023-04-03

**Authors:** Zhenxiang Zhan, Tong Xu, Ye Xu, Fangwang Fu, Zicheng Cheng, Lingfan Xia, Yucong Wu, Xuan Xu, Yungang Cao, Zhao Han

**Affiliations:** ^1^Department of Neurology, The Second Affiliated Hospital and Yuying Children's Hospital of Wenzhou Medical University, Wenzhou, China; ^2^Department of Neurology, Affiliated Jinhua Hospital, Zhejiang University School of Medicine, Jinhua, China

**Keywords:** acute ischemic stroke, intravenous thrombolysis, hemorrhagic transformation, brain atrophy, cerebral small vessel disease, leukoaraiosis

## Abstract

**Background:**

Hemorrhagic transformation (HT) is common among acute ischemic stroke patients after treatment with intravenous thrombolysis (IVT). We analyzed potential relationships between markers of cerebral small vessel disease (CSVD) and HT in patients after IVT.

**Methods:**

This study retrospectively analyzed computed tomography (CT) data for acute ischemic stroke patients before and after treatment with recombinant tissue plasminogen activator at a large Chinese hospital between July 2014 and June 2021. Total CSVD score were summed by individual CSVD markers including leukoaraiosis, brain atrophy and lacune. Binary regression analysis was used to explore whether CSVD markers were related to HT as the primary outcome or to symptomatic intracranial hemorrhage (sICH) as a secondary outcome.

**Results:**

A total of 397 AIS patients treated with IVT were screened for inclusion in this study. Patients with missing laboratory data (*n* = 37) and patients treated with endovascular therapy (*n* = 42) were excluded. Of the 318 patients included, 54 (17.0%) developed HT within 24–36 h of IVT, and 14 (4.3%) developed sICH. HT risk was independently associated with severe brain atrophy (OR 3.14, 95%CI 1.43–6.92, *P* = 0.004) and severe leukoaraiosis (OR 2.41, 95%CI 1.05–5.50, *P* = 0.036), but not to severe lacune level (OR 0.58, 95%CI 0.23–1.45, *P* = 0.250). Patients with a total CSVD burden ≥1 were at higher risk of HT (OR 2.87, 95%CI 1.38–5.94, *P* = 0.005). However, occurrence of sICH was not predicted by CSVD markers or total CSVD burden.

**Conclusion:**

In patients with acute ischemic stroke, severe leukoaraiosis, brain atrophy and total CSVD burden may be risk factors for HT after IVT. These findings may help improve efforts to mitigate or even prevent HT in vulnerable patients.

## Introduction

Ischemic strokes account for 71% of strokes worldwide (1). The preferred treatment for patients with acute ischemic stroke (AIS) is intravenous thrombolysis (IVT) using recombinant tissue plasminogen activator within 4.5 h of onset (2, 3). However, such IVT may cause hemorrhagic transformation (HT), which impedes functional recovery and can even lead to death (4). About 90% of HT cases occur within 24–36 h of stroke onset (5). Some factors have been associated with the development of HT, such as old age (6, 7), hypertension (7), atrial fibrillation (8), cerebral amyloid angiopathy (9–11) and high National Institutes of Health Stroke Scale (NIHSS) score (12, 13).

Cerebral small vessel disease (CSVD) is a disorder involving the brain's small perforating arterioles, capillaries and venules, and is attracting much interest (14). It leads to serious deterioration of cognitive function, gait and balance (15), and may worsen prognosis of AIS patients after IVT (15). A meta-analysis also linked HT risk to the presence of CSVD (16). Magnetic resonance imaging is the gold standard for diagnosing and assessing CSVD (17), but computed tomography (CT) is routinely used in many acute stroke units to identify patients suitable for acute treatments such as IVT. Computed tomography can assess several CSVD markers, including leukoaraiosis, lacune and brain atrophy (17). These markers may be useful for identifying patients at higher risk of intracerebral hemorrhage (18). Although magnetic resonance imaging is typically used to evaluate CSVD markers, CT offers faster assessment, which can be critical in time-sensitive situations such as IVT for AIS (19).

Studies about the association between CSVD markers based on CT and HT are rare. Thus we investigated the relationship between CSVD markers as detected by CT at stroke onset and risk of HT after IVT treatment.

## Methods

### Patients

This retrospective, observational, single-center study continuously collected clinical data of AIS patients treated with recombinant tissue plasminogen activator in the emergency department of the Second Affiliated Hospital of Wenzhou Medical University from July 2014 to June 2021. This study was approved by the ethics committee of the Second Affiliated Hospital and Yuying Children's Hospital of Wenzhou Medical University, which waived the requirement for written informed consent because the patients or their legal guardians, at the time of treatment, consented for their anonymized medical data to be analyzed and published for research purposes.

Patients were included in the study if they had been diagnosed with ischemic stroke according to the Chinese guidelines for the diagnosis and treatment of acute ischemic stroke, received 0.9 mg/kg of recombinant tissue plasminogen activator within 4.5 h of stroke onset, and underwent head CT before IVT at admission and within 24–36 h after IVT. The timeframe of 24–36 h after IVT was selected based on the European Cooperative Acute Stroke Study (20).

Patients were excluded if they were diagnosed with other central nervous system diseases, such as epilepsy, dementia, or Parkinson's disease; diagnosed with serious systemic diseases; or treated with endovascular therapy after IVT.

### Data collection

In addition to sex and age, the following data were extracted from electronic medical records: past medical history, including hypertension, diabetes, hyperlipidemia, atrial fibrillation, coronary heart disease, previous stroke history, smoking and drinking history, and previous antithrombotic drug treatment history; clinical parameters, such as systolic and diastolic blood pressure, NIHSS score, and time from onset to thrombolytic therapy; as well as laboratory data, such as venous blood glucose level at admission, international normalized ratio (INR), activated partial thrombin time (APTT), platelet count, total cholesterol and low-density lipoprotein cholesterol.

### CSVD assessment

Two experienced neurologists (ZZ, TX), who were blinded to other clinical information and who worked independently from each other, retrospectively assessed CSVD markers from CT scans. Discrepancies between investigators were addressed with a third investigator (ZH) joining the discussion. Disputes were resolved through discussion. Leukoaraiosis was scored according to the 3-point van Swieten scale (21), in which 0 points were given if no imaging evidence was observed, 1 point if the lesions were limited to the lateral ventricle, or 2 points if the lesions had spread from the lateral ventricle to the cerebral cortex.

Brain atrophy was classified as central or cortical, and its severity was assessed as none (0 point), mild to moderate (1 point) or severe (2 points) (22). We defined a “lacuna of presumed vascular origin” to be a round or ovoid, subcortical cavity of diameter 3–15 mm that was filled with a fluid similar in appearance to cerebrospinal fluid (19).

**Figure 1:**
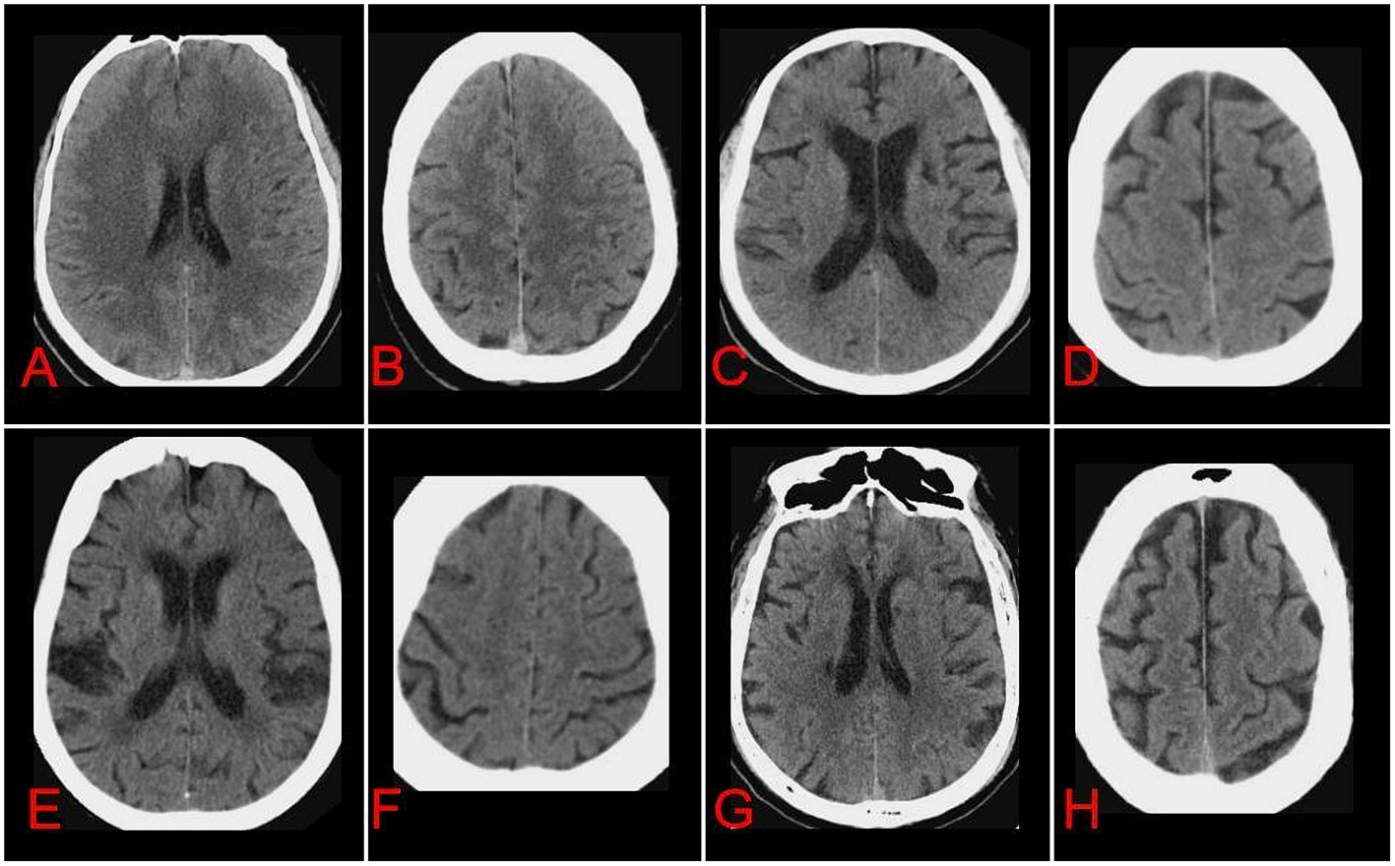
Axial non-contrast-enhanced CT images of brains from patients after acute ischemic stroke at the level of choroid plexus **(A, C, E, G)** or semioval center **(B, D, F, H)** of the lateral ventricle. Cerebral small-vessel disease (CSVD) markers were individually assessed and graded according to the appropriate scales, and total CSVD burden was calculated. Images in **(A, B)** are representative of patients with total CSVD burden of 0: anterior leukoaraiosis = 0, posterior leukoaraiosis = 0, no lacune, central atrophy = 0, cortical atrophy = 0. Images in **(C, D)** are representative of patients with total CSVD burden of 1: anterior leukoaraiosis = 1, posterior leukoaraiosis = 1, no lacune, central atrophy = 2, cortical atrophy = 1. Images in **(E, F)** are representative of patients with total CSVD burden of 2: anterior leukoaraiosis = 2, posterior leukoaraiosis = 2, no lacune, central atrophy = 2, cortical atrophy = 1. Images in **(G, H)** are representative of patients with total CSVD burden of 3: anterior leukoaraiosis = 2, posterior leukoaraiosis = 1, lacune in left and right basal ganglia = 2, central atrophy = 2, cortical atrophy = 2.

The scores for these individual CSVD markers were summed to a total CSVD score (23), meaning that 1 point for each of the following situations exist: a van Swieten score of 2; presence of at least 2 lacune; a central or cortical brain atrophy score of 2. Thus, the total CSVD burden could range from 0 (no imaging features of severe CSVD) to 3 (each imaging feature of CSVD was serious) (Figure 1).

### Primary and secondary outcomes

The same two neurologists also independently evaluated the CT images for the presence of HT as a primary outcome, which encompasses all types of post-ischemic hemorrhages. HT was further classified as hemorrhagic infarction (HI) types I and II and parenchymal hemorrhage (PH) types I and II. HI I is defined as small petechiae along the margins of the infarct, while HI II represents more confluent petechiae within the infarcted area, but without space-occupying effect. PH I is defined as blood clot not exceeding 30% of the infarcted area with some mild space-occupying effect, and PH II represents dense blood clot(s) exceeding 30% of the infarct volume with significant space-occupying effect (20).

The secondary outcome was symptomatic intracerebral hemorrhage (sICH), which was defined as bleeding visible anywhere on the cranial scan, accompanied by clinical deterioration or adverse events, such as drowsiness or aggravation of hemiplegia, leading to an increase in NIHSS score ≥4 points (24).

### Statistical analysis

Categorical variables were reported as frequencies and percentages, while continuous variables were reported as mean and standard deviation if the data were normally distributed, or as median and interquartile range if the data were skewed. Pearson's chi-squared test or Fisher's precision probability test was used for unordered categorical variables, while the Mann-Whitney U test was used if the data were continuous.

Binary regression analysis was used for two multivariable models in which variables with *P* < 0.1 after univariate analysis were analyzed further to determine risk factors independently related to HT or sICH. In the first model, the three components of total CSVD score (severe anterior or posterior leukoaraiosis, severe central or cortical atrophy, and severe lacune level) were treated as separate covariates. In the second model, the total burden of CSVD was dichotomized into 0 or ≥1 to evaluate its potential association with risk of HT. Risk of HT was expressed in terms of adjusted odds ratios (ORs) and corresponding 95% confidence intervals (CIs). Differences associated with two-sided *P* < 0.05 were considered statistically significant. All statistical analyses were performed using SPSS 24.0 (IBM, Armonk, NY, USA).

## Results

### Clinicodemographic characteristics

**Figure 2:**
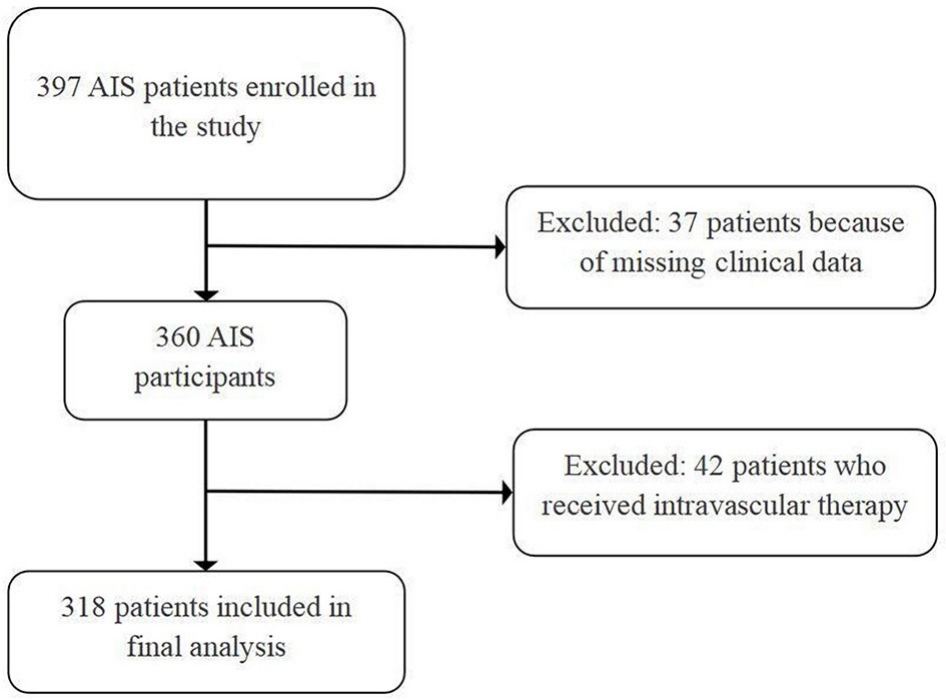
Flow diagram of patient enrollment. AIS, acute ischemic stroke.

A total of 397 AIS patients treated with IVT were screened for inclusion in this study. After excluding patients with missing laboratory data (*n* = 37) and patients treated with endovascular therapy (*n* = 42), 318 patients (66.4% men) were included in the final analysis (Figure 2).

Excellent inter-rater agreement for CSVD rating (κ = 0.83) was observed. The included sample had a median age of 70 years (interquartile range 61–80, Table 1). Fifty-four (17.0%) patients developed HT within 24–36 h after IVT. HT was significantly more frequent among patients with atrial fibrillation (66.7% vs. 25.4%, *P* < 0.001), higher median NIHSS score (11 vs. 6, *P* < 0.001), higher median baseline diastolic blood pressure (92 vs. 87, *P* = 0.045) and higher median INR (1.04 vs. 1.02, *P* = 0.007). And TOAST classification also had statistical significance (*P* < 0.001).

**Table 1:** Clinicodemographic characteristics of acute ischemic stroke patients^a^.

**Characteristic**	**HT (*n* = 54)**	**No HT (*n* = 264)**	***P***
Age (years)	76 (63–83)	70 (60–79)	**0.016**
Sex (female)	23 (42.5)	84 (31.8)	0.127
Hypertension	43 (79.6)	201 (76.1)	0.581
Diabetes mellitus	14 (25.9)	85 (32.2)	0.365
Hyperlipidemia	15 (27.7)	110 (41.7)	0.057
Atrial fibrillation	36 (66.7)	67 (25.4)	**<0.001**
Coronary heart disease	5 (9.2)	24 (9.1)	0.969
Previous stroke history	6 (11.1)	35 (13.3)	0.669
Smoking history	8 (14.8)	66 (25.0)	0.107
Drinking history	13 (24.0)	55 (20.8)	0.597
Previous antithrombotic therapy	6 (11.1)	33 (12.5)	0.777
Time from onset to thrombolysis (min)	160 (120–193)	170 (120–210)	0.411
NIHSS score (points)	11 (7–17)	6 (4–10)	**<0.001**
Systolic blood pressure level (mmHg)	162 (141–180)	159 (142–173)	0.360
Diastolic blood pressure (mmHg)	92 (81–104)	87 (78–97)	**0.045**
Blood glucose level (mmol/L)	7.3 (6.1–9.4)	7.0 (6.0–8.8)	0.345
Platelet count (10^9^/L)	181 (155–216)	190 (165–227)	0.066
INR	1.04 (1.01–1.10)	1.02 (0.97–1.09)	**0.007**
APTT (sec)	33.8 (31.2–36.7)	33.9 (31.0–36.8)	0.802
Total cholesterol (mmol/L)	4.3 (3.7–4.8)	4.4 (3.7–5.1)	0.419
Low-density lipoprotein cholesterol (mmol/L)	2.5 (1.9–3.1)	2.6 (2.0–3.3)	0.409
TOAST classification			**<0.001**
Large artery atherosclerosis	14 (25.9)	76 (28.8)	
Cardioembolism	31 (57.4)	70 (26.5)	
Small-artery occlusion	4 (7.4)	75 (28.4)	
Stroke of other determined cause	2 (3.7)	5 (1.9)	
Stroke of undetermined cause	3 (5.6)	38 (14.4)	
**Anterior leukoaraiosis**			
0	20 (37.0)	139 (52.7)	**0.010**
1	25 (46.3)	103 (39.0)	
2	9 (16.7)	22 (8.3)	
**Posterior leukoaraiosis**			
0	23 (42.6)	172 (65.2)	**0.001**
1	21 (38.9)	61 (23.1)	
2	10 (18.5)	31 (11.7)	
Severe anterior or posterior leukoaraiosis	14 (25.9)	40 (15.2)	**0.002**
**Cortical atrophy**			
0	6 (11.1)	65 (24.6)	**<0.001**
1	29 (53.7)	166 (62.9)	
2	19 (35.2)	33 (12.5)	
**Central atrophy**			
0	10 (18.5)	106 (40.2)	**<0.001**
1	27 (50.0)	129 (48.9)	
2	17 (31.5)	29 (11.0)	
Severe cortical or central atrophy	26 (48.1)	45 (17.0)	**<0.001**
**Lacune**			
0	28 (51.9)	142 (53.8)	0.984
1	17 (25.9)	70 (26.5)	
≥2	9 (22.2)	52 (19.7)	
**Total CSVD score**			
0	19 (35.2)	166 (62.9)	**<0.001**
1	19 (35.2)	62 (23.5)	
2	14 (25.9)	33 (12.5)	
3	2 (3.7)	3 (1.1)	
Total CSVD score ≥ 1	35 (64.8)	98 (37.1)	**<0.001**

Values are n (%) or median (interquartile range), unless otherwise noted. Boldfaced values differ significantly between the two groups.

a Mann-Whitney U test.

HT, hemorrhagic transformation; APTT, activated partial thromboplastin time; INR, international normalized ratio; NIHSS, National Institutes of Health Stroke Scale; CSVD, cerebral small vessel disease.

### Associations of CSVD markers with HT

Compared to patients who did not suffer from HT, patients with HT showed higher incidence of severe anterior and posterior leukoaraiosis, severe central and cortical atrophy, and greater total CSVD burden (Tables 2, 3). There was no significant difference in lacune level between HT and non-HT patients (*P* = 0.984).

**Table 2:** Regression analysis to identify associations of clinicodemographic characteristics with risk of hemorrhagic transformation after intravenous thrombolysis^a^.

**Characteristic**	**OR (95%CI)**	***p***
Age	0.97 (0.94–1.00)	0.067
Atrial fibrillation	5.11 (2.32–11.24)	**<0.001**
Baseline NIHSS score	1.05 (1.00–1.10)	**0.035**
Baseline diastolic blood pressure	1.00 (0.98–1.02)	0.772
Platelet count	0.99 (0.99–1.00)	0.518
Hyperlipidemia	0.62 (0.30–1.29)	0.205
INR	0.16 (0.01–4.58)	0.286
Severe leukoaraiosis (≥2)	2.41 (1.05–5.50)	**0.036**
Severe brain atrophy (≥2)	3.14 (1.43–6.92)	**0.004**
Severe lacune level (≥2)	0.58 (0.23–1.45)	0.250

a Adjusted for individual scores for leukoaraiosis, brain atrophy, or lacune.

Boldfaced values indicate statistically significant independent risk factors.

OR, odds ratio; CI, confidence interval; INR, international normalized ratio; NIHSS, National Institutes of Health Stroke Scale.

**Table 3:** Regression analysis to identify associations of clinicodemographic characteristics with risk of hemorrhagic transformation after intravenous thrombolysis^a^.

**Characteristic**	**OR (95%CI)**	***p***
Age	0.97 (0.94–1.00)	0.144
Atrial fibrillation	5.52 (2.51–12.15)	**<0.001**
Baseline NIHSS score	1.05 (1.00–1.10)	**0.044**
Baseline diastolic blood pressure	1.00 (0.98–1.02)	0.507
Platelet count	0.99 (0.99–1.00)	0.534
Hyperlipidemia	0.60 (0.29–1.24)	0.173
INR	0.41 (0.01–10.78)	0.594
Total CSVD score ≥ 1	2.87 (1.38–5.94)	**0.005**

a Adjusted for total cerebral small vessel disease burden.

Boldfaced values indicate statistically significant independent risk factors.

OR, odds ratio; CI, confidence interval; INR, international normalized ratio; NIHSS, National Institutes of Health Stroke Scale; CSVD, cerebral small vessel disease.

Risk of HT was independently related to severe brain atrophy (OR 3.14, 95%CI 1.43–6.92, *P* = 0.004) and severe leukoaraiosis scores (OR 2.41, 95%CI 1.05–5.50, *P* = 0.036), but not to severe lacune level (OR 0.58, 95%CI 0.23–1.45, *P* = 0.250). A total CSVD burden of ≥1 greatly increased risk of HT (OR 2.87, 95%CI 1.38–5.94, *P* = 0.005). According to either regression model, risk of HT was also higher in the presence of atrial fibrillation or high baseline NIHSS score.

### Associations of CSVD markers with secondary outcomes

Fourteen (4.3%) patients experienced sICH after IVT, and those who did or did not experience sICH did not differ significantly in severe leukoaraiosis, brain atrophy or lacune level (Supplementary Table S1). Total CSVD burden ≥1 was not associated with greater risk of sICH after IVT. Atrial fibrillation, in contrast, was more frequent among patients with sICH (71.4% vs. 30.6%, *P* = 0.001, Supplementary Table S1) and it predicted sICH risk even after adjusting for individual CSVD markers (OR 4.65, 95%CI 1.27–16.95, *P* = 0.020) or adjusting for total CSVD burden (OR 4.23, 95%CI 1.20–14.88, *P* = 0.024; Supplementary Tables S2, S3).

## Discussion

To our knowledge, this is the first retrospective study to investigate the relationship between CSVD markers, as visualized by CT, and risk of HT in AIS patients after IVT. Our results suggest that severe leukoaraiosis, severe brain atrophy and total CSVD burden in AIS patients prior to IVT are independently associated with increased risk of HT after IVT.

Several studies have already shown strong links between presence of leukoaraiosis and hemorrhage after IVT, but the reasons for this correlation are unclear (16, 25–28). Leukoaraiosis is thought to reflect ischemic white matter injury penetrating small vessels in the distal deep artery or arteriole area, which may reflect chronic endothelial dysfunction (29). At the same time, leukoaraiosis may increase small vessel brittleness and vascular rupture, leading to bleeding complications and breakdown of the blood-brain barrier (29, 30). Inflammation in the central nervous system or its periphery as well as variations in blood pressure may also contribute to leukoaraiosis pathogenesis (31–36).

Similar to leukoaraiosis, brain atrophy has also been associated with HT, though the mechanisms are unclear. One possibility is that brain atrophy in AIS patients at least partly reflects pre-existing brain injury due, for example, to degenerative processes in dementia or subcortical vascular encephalopathy (37). Our study is consistent with earlier work showing that brain atrophy and other markers may reflect risk of intracerebral hemorrhage (38). In contrast, we did not find a significant relationship between lacune level and risk of HT, similar to the few other studies that have examined this question (39, 40).

It is clear that there is no single pathological mechanism explaining how leukoaraiosis or brain atrophy leads to HT after IVT. We found that the aggregate parameter of total CSVD burden, when it was 1 or higher, predicted higher risk of HT after IVT. Total CSVD burden provides a more complete view of the impact of CSVD on the brain, more so than individual CSVD features, and it may be easier to use in the clinic. It may also facilitate comparisons between medical centers, so long as the definitions of single CSVD features are standardized (19). Arguments could be made that the potential differences in the pathogenic mechanisms leading to these different CSVD features may render an aggregate index unreliable. However, we would reason that leukoaraiosis, brain atrophy, and lacune are all consequences of small vessel diseases and often occur simultaneously, allowing their aggregation into a single, more practical index that does not make detailed assumptions about specific pathogenic mechanisms. The reliability and accuracy of total CSVD burden for predicting HT risk should be examined further in larger studies and benchmarked against magnetic resonance imaging.

We did not find that CSVD markers alone or total CSVD burden predicted increased risk of sICH. A meta-analysis of 13 studies found that presence of leukoaraiosis or total CSVD burden increased risk of sICH after IVT (16). However, only two of those individual studies evaluated CSVD markers through CT. The remaining studies used magnetic resonance imaging because it is better at detecting cerebral microbleeds, which are closely related to sICH (18). This may help explain why we failed to detect correlations between CSVD markers and sICH. Another obstacle to comparing our findings to the literature is that different studies can define sICH differently (28, 41).

This study has some limitations. First, our study was retrospective and included a small sample from a single center. Second, we did not collect additional data relevant to prognosis, such as the modified Rankin scale score after 3 months, which may help clarify relationships between CSVD markers and HT.

Despite these limitations, our results suggest that AIS patients with severe leukoaraiosis, severe brain atrophy and total CSVD burden >1 based on CT before IVT are at increased risk of HT after IVT. These parameters might be useful markers to identity patients with high risk of HT and assist the treatment decision-making, thus it should be verified and further explored in larger, prospective studies.

## Data availability statement

The raw data supporting the conclusions of this article will be made available by the authors, without undue reservation.

## Ethics statement

Ethical review and approval was not required for the study on human participants in accordance with the local legislation and institutional requirements. Written informed consent for participation was not required for this study in accordance with the national legislation and the institutional requirements.

## Author contributions

ZZ, TX, and ZH conceived and designed the study. TX, FF, ZC, LX, YW, and YC performed experiments. ZZ analyzed data. ZZ and ZH wrote the manuscript. All authors revised the manuscript and approved the final version.
