# Supplementary material for: Associations between computed tomography markers of cerebral small vessel disease and hemorrhagic transformation after intravenous thrombolysis in acute ischemic stroke patients
Source: Front Neurol. 2023 Apr 3;14:1144564. doi: 10.3389/fneur.2023.1144564 (PMC10106596; doi:10.3389/fneur.2023.1144564)
Supplement: Supplementary file 1 [file Data_Sheet_1.docx]

**Supplementary tables**

**Table S1:** Clinicodemographic data of acute ischemic stroke patients stratified by occurence of symptomatic intracerebral hemorrhage

| **Characteristic** | **sICH**  **(n=14)** | **No sICH**  **(n=304)** | ***p*** |
| --- | --- | --- | --- |
| Age (years) | 61 (53-80) | 70 (61-80) | 0.375 |
| Sex (female) | 6 (42.9) | 101 (33.2) | 0.456 |
| Hypertension | 10 (71.4) | 234 (77.0) | 0.632 |
| Diabetes mellitus | 3 (21.4) | 96 (31.6) | 0.423 |
| Hyperlipidemia | 4 (28.6) | 121 (39.8) | 0.401 |
| Atrial fibrillation | 10 (71.4) | 93 (30.6) | **0.001** |
| Coronary heart disease | 0 (0) | 29 (9.5) | 0.226 |
| Previous stroke history | 1 (7.1) | 40 (13.2) | 0.512 |
| Smoking history | 3 (21.4) | 71 (23.4) | 0.868 |
| Drinking history | 4 (28.6) | 64 (21.1) | 0.503 |
| Previous antithrombotic therapy | 1 (7.1) | 38 (12.5) | 0.551 |
| Time from onset to thrombolysis (min) | 170 (120-205) | 166 (120-210) | 0.836 |
| NIHSS score (points) at baseline | 8 (4-11) | 7 (4-12) | 0.780 |
| Systolic blood pressure level (mmHg) at baseline | 165 (135-183) | 159 (142-174) | 0.673 |
| Diastolic blood pressure (mmHg) at baseline | 94 (81-110) | 87 (78-98) | 0.057 |
| Blood glucose level (mmol/L) at baseline | 7.5 (6.4-9.2) | 7.0 (6.0-8.8) | 0.725 |
| Platelet count (10^9^ /L) | 170 (124-202) | 190 (164-227) | 0.085 |
| INR | 1.07 (1.01-1.11) | 1.03 (0.98-1.09) | 0.146 |
| APTT (seconds) | 32.2 (30.5-34.7) | 33.9 (31.1-36.8) | 0.192 |
| Total cholesterol (mmol/L) | 3.9 (3.1-4.3) | 4.4 (3.7-5.1) | 0.056 |
| Low-density lipoprotein cholesterol (mmol/L) | 2.4 (1.7-3.2) | 2.6 (2.0-3.3) | 0.334 |
| TOAST classification |  |  |  |
| Large artery atherosclerosis | 3 (21.4) | 87 (28.6) | 0.539 |
| Cardioembolism | 6 (42.9) | 95 (31.3) |  |
| Small-artery occlusion | 2 (14.3) | 77 (25.3) |  |
| Stroke of other determined cause | 1 (7.1) | 6 (2.0) |  |
| Stroke of undetermined cause | 2 (14.3) | 39 (12.8) |  |
| Anterior leukoaraiosis |  |  |  |
| 0 | 8 (57.1) | 150 (49.3) | 0.744 |
| 1 | 4 (28.6) | 125 (41.1) |  |
| 2 | 2 (14.3) | 29 (9.5) |  |
| Posterior leukoaraiosis |  |  |  |
| 0 | 9 (64.3) | 186 (61.2) | 0.688 |
| 1 | 4 (28.6) | 74 (24.3) |  |
| 2 | 1 (7.1) | 44 (14.5) |  |
| Severe anterior or posterior leukoaraiosis | 3 (21.4) | 55 (18.1) | 0.752 |
| Cortical atrophy |  |  |  |
| 0 | 3 (21.4) | 68 (22.4) | 0.435 |
| 1 | 7 (50.0) | 188 (61.8) |  |
| 2 | 4 (28.6) | 48 (15.8) |  |
| Central atrophy |  |  |  |
| 0 | 6 (42.9) | 110 (36.2) | 0.940 |
| 1 | 5 (35.7) | 151 (49.7) |  |
| 2 | 3 (21.4) | 43 (14.1) |  |
| Severe cortical or central atrophy | 4 (28.6) | 67 (22.0) | 0.567 |
| lacune |  |  |  |
| 0 | 6 (42.9) | 164 (53.9) | 0.482 |
| 1 | 5 (35.7) | 82 (27.0) |  |
| ≥ 2 | 3 (21.4) | 58 (19.1) |  |
| Total CSVD score |  |  |  |
| 0 | 8 (57.1) | 177 (58.2) | 0.775 |
| 1 | 3 (21.4) | 78 (25.7) |  |
| 2 | 2 (14.3) | 45 (14.8) |  |
| 3 | 1 (7.1) | 4 (1.3) |  |
| Total CSVD score ≥ 1 | 6 (42.9) | 127 (41.8) | 0.936 |

Values are n (%) or median (interquartile range), unless otherwise noted.

Boldfaced values differ significantly between the the two groups.

The boldfaced P value was significant (*p* < 0.05).

^a^ Mann-Whitney U test.

Abbreviations: sICH, symptomatic intracerebral hemorrhage; APTT, activated partial thromboplastin time; INR, international normalized ratio; NIHSS, National Institutes of Health Stroke Scale; CSVD, cerebral small vessel disease.

**Table S2:** Regression analysis to identify associations between clinical characteristics and sICH after intravenous thrombolysis ^a^

| **Characteristic** | | **OR（95%CI）** | ***p*** |
| --- | --- | --- | --- |
| Atrial fibrillation | 4.65 (1.27-16.95) | **0.020** |  |
| Baseline diastolic blood pressure | 1.02 (0.99-1.05) | 0.085 |  |
| Platelet count | 0.99 (0.98-1.00) | 0.530 |  |
| Total cholesterol | 0.60 (0.32-1.13) | 0.116 |  |
| Severe leukoaraiosis (≥ 2) | 0.83 (0.20-3.45) | 0.800 |  |
| Severe brain atrophy (≥ 2) | 0.57 (0.14-2.32) | 0.440 |  |
| Severe lacune level (≥ 2) | 1.49 (0.36-6.13) | 0.573 |  |

^a^ Adjusted for individual scoring of leukoaraiosis, brain atrophy, or lacune.

Boldfaced values indicate statistically significant independent risk factors.

Abbreviations: sICH, symptomatic intracerebral hemorrhage; OR, odds ratio; CI, confidence interval.

**Table S3:** Regression analysis to identify associations between clinical characteristics and sICH after intravenous thrombolysis ^a^

| **Characteristic** | **OR（95%CI）** | ***p*** |
| --- | --- | --- |
| Atrial fibrillation | 4.23 (1.20-14.88) | **0.024** |
| Baseline diastolic blood pressure | 1.02 (0.99-1.05) | 0.093 |
| Platelet count | 0.99 (0.98-1.00) | 0.521 |
| Total cholesterol | 0.61 (0.32-1.14) | 0.124 |
| CSVD total score≥1 | 0.63 (0.19-2.07) | 0.455 |

^a^ Adjusted for total cerebral small vessel disease burden.

Boldfaced values indicate statistically significant independent risk factors.

Abbreviations: sICH, symptomatic intracerebral hemorrhage; OR, odds ratio; CI, confidence interval; CSVD, cerebral small vessel disease.
